# ‘It's really important to be collaborating’: Experiences of participatory research for Chinese and Vietnamese parents of autistic children

**DOI:** 10.1177/23969415231210482

**Published:** 2023-11-13

**Authors:** Jodie Smith, Aspasia Stacey Rabba, Poulomee Datta, Emma Dresens, Rena Wang, Lin Cong, Ngoc Dang, Gabrielle Hall, Melanie Heyworth, Wenn Lawson, Patricia Lee, Rozanna Lilley, Emily Ma, Hau T T Nguyen, Kim-Van Nguyen, Phuc Nguyen, Chong Tze Yeow, Elizabeth Pellicano

**Affiliations:** Macquarie School of Education, 7788 Macquarie University, Sydney, Australia; School of Allied Health, Human Services and Sport, 2080 La Trobe University, Melbourne, Australia; Department of Psychology, Counselling and Therapy, School of Psychology and Public Health, 2080 La Trobe University, Melbourne, Australia; Macquarie School of Education, 7788 Macquarie University, Sydney, Australia; Department of Psychology, Counselling and Therapy, School of Psychology and Public Health, 2080 La Trobe University, Melbourne, Australia; Faculty of Education, School of Educational Psychology and Counselling, 2541 Monash University, Melbourne, Australia; Macquarie School of Education, 7788 Macquarie University, Sydney, Australia; Positive Partnerships, Chatswood, NSW, Australia; Macquarie School of Education, 7788 Macquarie University, Sydney, Australia; Department of Psychology, Counselling and Therapy, School of Psychology and Public Health, 2080 La Trobe University, Melbourne, Australia; Macquarie School of Education, 7788 Macquarie University, Sydney, Australia; Macquarie School of Education, 7788 Macquarie University, Sydney, Australia; Macquarie School of Education, 7788 Macquarie University, Sydney, Australia; Macquarie School of Education, 7788 Macquarie University, Sydney, Australia; Reframing Autism, Sydney, Australia; Macquarie School of Education, 7788 Macquarie University, Sydney, Australia; Curtin University, Curtin Autism Research Group, Perth WA; Department of Psychology, Counselling and Therapy, School of Psychology and Public Health, 2080 La Trobe University, Melbourne, Australia; Positive Partnerships, Forestville, NSW, Australia; Positive Partnerships, Chatswood, NSW, Australia; Macquarie School of Education, 7788 Macquarie University, Sydney, Australia; Macquarie School of Education, 7788 Macquarie University, Sydney, Australia; Department of Psychology, Counselling and Therapy, School of Psychology and Public Health, 2080 La Trobe University, Melbourne, Australia; Macquarie School of Education, 7788 Macquarie University, Sydney, Australia; Macquarie School of Education, 7788 Macquarie University, Sydney, Australia; Macquarie School of Education, 7788 Macquarie University, Sydney, Australia; Macquarie School of Education, 7788 Macquarie University, Sydney, Australia; Macquarie School of Education, 7788 Macquarie University, Sydney, Australia; Department of Clinical, Educational and Health Psychology, 4919 University College London, London, UK

**Keywords:** Participatory research, community participation, cultural and linguistic diversity, autism, impact

## Abstract

**Background and aims:**

Participatory research involves academic partners working together with the community that is affected by research to make decisions about that research. Such approaches often result in research that is more respectful of, and responsive to, community preferences – and is vital in the context of autism research with culturally and linguistically diverse (CALD) communities. Whilst participatory approaches are becoming more commonplace within CALD autism research, no studies have explored the experiences of being involved in autism research from the perspectives of CALD community partners over the course of a study. This paper intended to address this gap by reporting on the experiences of CALD parents of autistic children who were community partners in a 1-year Australian research project exploring home–school partnerships for CALD parents of autistic children. We aimed to: (1) report on how parents’ involvement in the research process shaped the home–school partnerships study over time and (2) understand their experiences of being community partners on the home–school partnerships project.

**Methods:**

Using key principles of participatory approaches, we established Chinese and Vietnamese parent advisory groups to contribute to a project exploring home–school partnerships for parents of autistic children from CALD backgrounds in Australia. Advisory groups included parents of autistic children from Chinese/Vietnamese backgrounds, as well as interpreters, professionals and researchers. We documented how parents’ participation as community partners shaped the home–school partnerships study over the course of the project. We also elicited parents’ own views and experiences of being community partners through informal, open-ended questions at the beginning and end of the study.

**Results:**

We found that parents’ input fundamentally shaped the broader home–school partnership study, from meaningful, accurate translation of interview schedules through to making decisions regarding community-specific recommendations and dissemination plans. Parents themselves reported being keen to collaborate and to hear and share opinions for the purpose of the home–school partnership study – although they noted how emotionally difficult sharing their stories could be. While they initially had some concerns about combining being involved as a community partner with their existing responsibilities, ultimately, parents were surprised by the scope of the home–school partnership study and their level of involvement as community partners. Through hearing others’ stories and sharing their own in advisory group meetings, parents reported ancillary benefits of their involvement, including increased self-advocacy and well-being.

**Conclusions:**

These findings show how research that is conducted in partnership *with* diverse members of the autism community has the capacity to improve the quality of the research and benefit community partners.

**Implications:**

This study clearly documents the benefits and potential challenges of participatory approaches with CALD communities. These findings emphasise to researchers and funders the importance of including extra time and money within budgets in order to produce meaningful research that is respectful and responsive to communities.

## Introduction

Participatory or co-produced research supports collaboration between researchers, practitioners and community members (Hickey et al., 2018). This approach aims to produce studies that are respectful, ethical and responsive to the needs and preferences and places communities at the centre of the research (Collins et al., 2018). The advantages of participatory research are now well documented in the literature, with potential benefits including collaborative, sustained relationships between stakeholders, the co-learning and co-creation of knowledge and better interpretation and broader dissemination of research findings (Amauchi et al., 2021). As an illustration, participatory research with First Nations communities has highlighted how iterative discussions between community members and researchers during the co-production process can safeguard the accuracy of data interpretation and the relevancy of community-targeted messages (Kildea et al., 2009).

### Participatory research, autism and diverse communities

There has been a promising upsurge in co-produced autism research over the last decade (Beaumont, 2019; Fletcher-Watson et al., 2019; Poulsen et al., 2022), especially with autistic adults (Nicolaidis et al., 2019). More recently, there has been a smaller, but no less welcome, increase in co-produced autism research with culturally and linguistically diverse (CALD) communities. For example, recent Australian research studies have explored attitudes towards autism in Aboriginal and Torres Strait Islander communities using participatory approaches (Lilley et al., 2019, 2020, 2021), and participatory approaches have also been used in studies with Somali communities in the United Kingdom (Aabe et al., 2019; Fox et al., 2017; Selman et al., 2018).

These aforementioned studies in the field of autism research have described similar benefits reported in other CALD and non-CALD co-produced studies (De las Nueces et al., 2012; De Souza et al., 2021; Kildea et al., 2009; Wild et al., 2021) – namely more effective dissemination of community messages, improved trust and ongoing collaboration between researchers and community members and increased skills and expertise of all participants. For co-produced CALD autism research, other benefits have also been reported – benefits that extend beyond the research itself. For instance, following involvement in a participatory research project, CALD community members have described feeling encouraged to better challenge stigma around autism in their own communities (Aabe et al., 2019). Given that autism is often more stigmatised in non-Western communities (Kandeh et al., 2020), including in Asian communities (Ha & Whittaker, 2022; Liu & Fisher, 2017), such ancillary benefits to being involved in the research process might also extend to CALD community members.

### Evaluation of the participatory process

Although co-produced studies are growing momentum, key features of the participatory approach (i.e., advisory groups and community consultation) are often task-oriented so there may be limited dedicated time for shared reflection on community members’ involvement in the research process itself (Banks et al., 2014). As such, we have little knowledge on the actual process of conducting participatory research, even though respecting and evaluating the impact of co-produced research is a key aspect of the participatory research process (Hickey et al., 2018). Those few existing studies that have evaluated the participatory process after the research was concluded (den Houting et al., 2021, 2022; Jose et al., 2020) have found that, while stakeholders were broadly positive about their experiences, they remained unclear about their roles/responsibilities and found power imbalances between researchers and community members challenging.

These studies, while important, have all collected participants’ experiences about the research process once it has been completed. Yet, without pre-emptive reflections about the research process, we cannot understand what motivates community partners to engage in the participatory process in the first place, or indeed learn about their participation expectations. It also means that we cannot determine whether experiences and reflections change over the research journey for community partners, making it difficult to identify barriers and enablers to effective future research co-production with different communities (O’Brien et al., 2021; Pinto et al., 2013).

### The current study

The current study sought to address this gap by eliciting community members’ experiences of research involvement using a longitudinal design. The community members involved in this study were partners on a 1-year Australian research project exploring Chinese and Vietnamese parents’ experiences of home–school partnerships for their autistic children (see Smith et al., 2022, 2023). For that project, we had convened a Chinese parent advisory group and a Vietnamese parent advisory group, who worked together with a team of autistic and non-autistic researchers, and who brought specific experiential expertise to the project of living with, and caring for, autistic children in particular cultural communities. Our parent advisory group members met four times over the course of the year-long home–school partnerships study and provided input on all aspects of the research process, including development, implementation, interpretation and dissemination. During the first and final advisory group meetings, we also conducted group-based interviews to understand parents’ experiences of the participatory process over time.

Here, we report on the data from both the advisory group meetings and the group-based interviews to address the following aims: (1) to document the impact of parents’ involvement as partners in the research on the broader home–school partnership study and (2) to understand partners’ own experiences of being involved in the home–school partnerships project and its perceived impact over the course of the 1-year project.

## Methods

### Advisory group members

For this project, researchers partnered with Positive Partnerships, an organisation providing information and supports for parents, carers and educators of school-aged children on the autism spectrum. Positive Partnerships had pre-existing involvement and trusting relationships with Vietnamese and Chinese communities, and these relationships laid the foundation for our own participatory approach here. All Vietnamese and Chinese parents were recruited through a purposive, snowball approach whereby parents were approached via email or phone by someone known to them in preferred languages (i.e., an interpreter, another member of the community or a researcher). Parents were all based in Melbourne, Australia. The composition of the advisory groups and the way in which meetings were held were based on core principles of participatory approaches with CALD and autism communities (for more information, see Aabe et al., 2019; Fletcher-Watson et al., 2019; see also Supplemental Table 1), including all non-salaried members being remunerated for their direct and indirect contributions.

#### Chinese parents

Five parents participated in the Chinese parent advisory group meetings (four mothers and one father). They were aged between 34 and 57 years and spoke either Mandarin (*n *= 4) or Cantonese (*n* = 1) at home. Three Chinese parents had been involved as participants in a previous research project; the remaining two had no prior research experience. All parents had one autistic son. One child had completed his formal education in specialist settings. The remaining children were in kindergarten, mainstream prep (i.e., the first year of formal Australian schooling) and Grade 11 of a special education school.

#### Vietnamese parents

Three parents participated in the Vietnamese parent advisory group meetings (two mothers and one father). Vietnamese was the home language for all parents. Parents ranged between 40 and 44 years (*n* = 1, missing). None of the Vietnamese parents had previous research experience. Two parents had one autistic child (one son and one daughter) and one parent had three autistic daughters. Children were in mainstream settings at different year levels (preschool, Grades 2, 4 and 11). The remaining child had completed his formal education in specialist settings.

#### Other attendees

In addition to the Vietnamese and Chinese parent members, two of the broader research team [JS & ED] were core members of both the Chinese and the Vietnamese parent advisory group meetings. Trained interpreters who spoke Mandarin and Cantonese [PL] and Vietnamese [KVN] were also involved in the Chinese and the Vietnamese parent advisory group meetings, respectively. Both interpreters had experience working with families with autistic children and had strong links with their respective communities. Attendees from both advisory groups are co-authors of this paper.

### Procedure

#### Advisory group meetings

**Figure 1.**
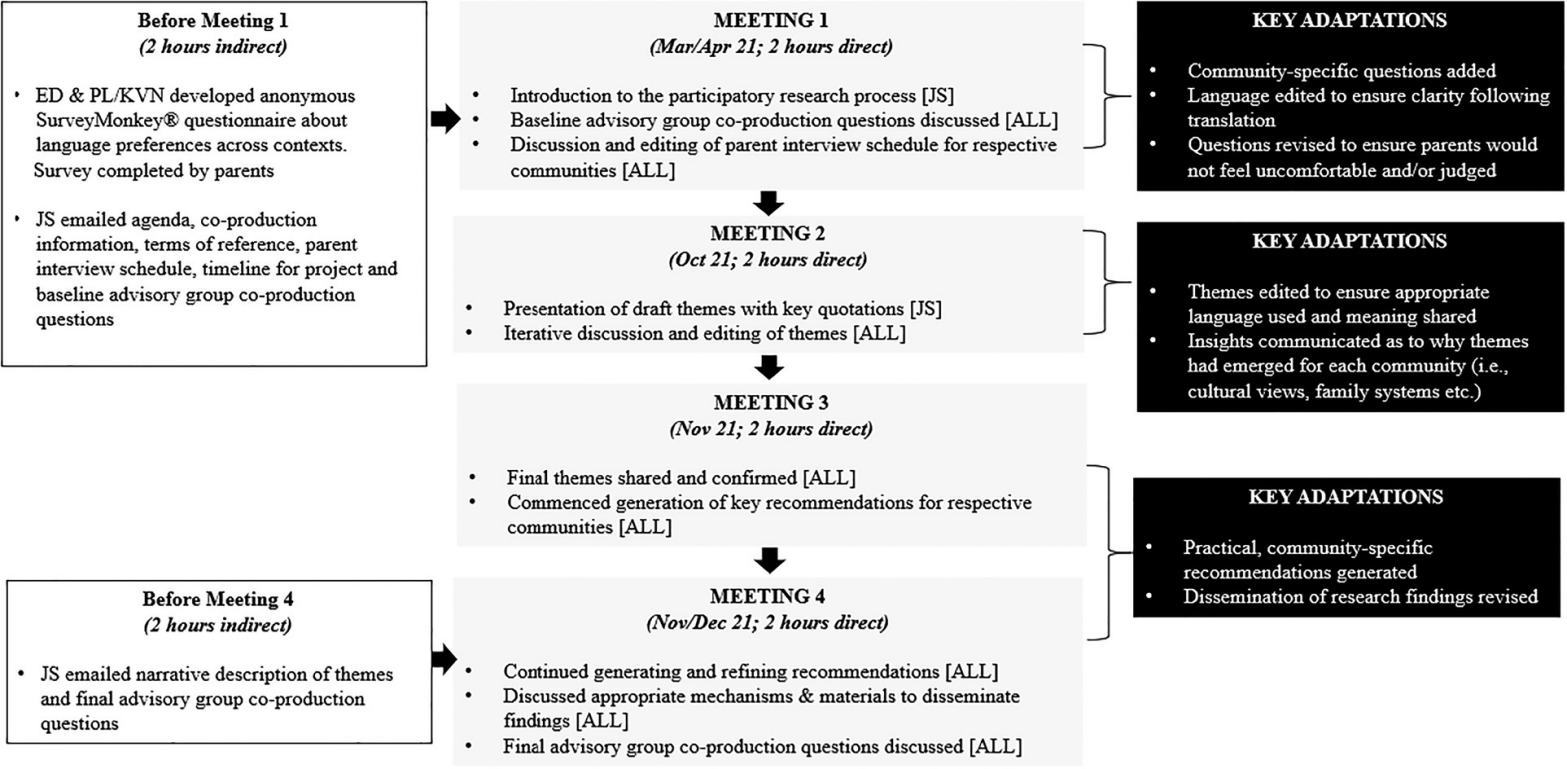
Meeting information and key adaptations to the research project following advisory group meetings.

Figure 1 presents advisory group meeting details and key adaptations to the broader research project that arose directly from advisory group meetings. Prior to the initial meeting with parents, we asked them to complete a brief, anonymous SurveyMonkey^®^ questionnaire about their written and spoken language preferences across contexts in both English and Simplified Chinese or Vietnamese. Results from this survey informed how the meetings were facilitated. All parents across both groups reported a preference for speaking in both English and home languages, so English was also the main language used during meetings. Interpreters were always present and parents were encouraged to speak in whatever language they preferred at each meeting (as per past guidance; see Zhang & Guttormsen, 2016).

For text-based documents, one Chinese respondent preferred Traditional Chinese and one Vietnamese respondent preferred English. Other respondents preferred reading in a mix of English and community languages. As such, pre-meeting coordination and documentation were also largely in English, although individual phone calls/emails were exchanged between some parents and interpreters to allow for any written information to be explained in home languages.

#### Meeting format

Both advisory group meetings followed the same process. Four, 2-hour advisory group meetings were held for each group between March and December 2021. All advisory group meetings were recorded and a summary of the discussion was disseminated immediately following the meeting. Meetings times varied (i.e., daytime vs. evenings) to accommodate parents’ availability. Due to the ongoing COVID-19 pandemic, advisory group meetings took place on Zoom and all four meetings were attended by all parents.

During the first advisory group meeting, the researcher [JS] briefly described the participatory research process with the bulk of the meeting spent discussing and editing proposed Chinese/Vietnamese parent interview questions for the home–school partnerships study. The second advisory group meeting occurred once all parent interviews had been completed and thematic analysis of interview transcripts for the home–school partnerships study had begun. In this second meeting, the researcher [JS] presented draft themes with key quotations. These themes were iteratively discussed and revised as a group. The final two advisory group meetings were devoted to the development of key recommendations from the home–school partnerships study for the specific communities and identification of the most suitable ways to disseminate the information.

### Reflections on expectations and experiences of the research process

To directly elicit parents’ views and experiences of participating in the home–school partnerships study, we embedded group-based interviews at the outset and completion of the broader research study. These interviews comprised several informal, open-ended questions based on past research using participatory approaches (Pinto et al., 2013; see Table 1 for specific questions). Specifically, interviews took place before the start of the first advisory group (47 min total) and before the end of the fourth and final advisory group (55 min total). Questions were designed to understand motivations, expectations and experiences of parents’ involvement in the advisory group meetings and, ultimately, of the participatory research process. Questions were emailed to all parents approximately 2 weeks ahead of the respective advisory group meetings to allow time to prepare answers and discuss questions with interpreters. Questions were posed in the group settings both to build rapport between parents, as well as to gather a richer understanding of divergent opinions, which can happen through the group discussion process (Merry et al., 2011). All advisory group members were present for these discussions. Discussions were recorded and subsequently transcribed verbatim for analysis. All parents verbally consented to contributing to this study.

**Table 1.** Questions asked of advisory group members (Pinto et al., 2013).

Baseline questions	Follow-up questions
1. Can you tell me about any experience you have in research (including co-production of research, like in this study)?	1. No question
2. What are your initial thoughts and feelings about collaborating in this research?	2. What are your final thoughts and feelings about collaborating in this research?
3. Can you tell me about any expectations you have about being part of this process?	3. Did the process meet your expectations you had at the beginning?
4. Can you tell me what you think you might learn during this process?	4. What did you learn during this process?
5. Can you tell me about any key challenges you might expect to face during this process	5. Were there any key challenges for you during this process?

### Data analysis

Before data were analysed, transcriptions were checked for accuracy with imprecisions corrected and filler words/repetitions deleted for comprehensibility. We followed Braun and Clarke's (2006, 2019) reflexive thematic analysis method. We adopted an inductive (bottom-up) approach (i.e., without integrating the themes within any pre-existing coding schemes or preconceptions of the researchers) to identify patterned meanings within the dataset. Our epistemological stance fits within an essentialist framework, in which we report the experiences, meanings, and reality of the participants (Byrne, 2021). We analysed the Chinese and Vietnamese transcripts both cross-sectionally (separately after each advisory group meeting) and longitudinally. Our longitudinal approach to analysis sought to avoid a descriptive analysis of the cross-sectional data (i.e., what happened at each time point), and instead brought the cross-sectional analyses together to focus on the *changes* between time points (Calman et al., 2013).

To begin, one senior researcher [JS] immersed herself in the transcripts/data, taking notes on striking and recurring observations and using NVivo to apply codes to each transcript. Meetings with SR and EP were used to discuss transcripts, reflect on ‘analytic noticings’ and explore alternate interpretations of, or ideas about, the data and subsequent generation of codes (Byrne, 2021). Next, JS, SR and EP generated a draft thematic map showing potential themes and subthemes. This map, along with all supporting quotes, was discussed at length with the broader team. The final themes and subthemes, which focused on the semantic features of the data (ensuring we stayed close to parents’ own language), were therefore identified through systematic engagement with the data combined with an active and deeply reflexive approach to analysis, influenced by the researchers’ and parent community members’ own aims and interpretation of the data (Braun & Clarke, 2006, 2019).

### Ethics

Ethical approval was obtained for the broader study (Macquarie University HREC Ref. No.: 5202196412836) and work was conducted in accordance with the Declaration of Helsinki (World Medical Association, 2013).

## Results

The results section begins by reporting, first, on how parents’ involvement shaped the broader study (Aim 1). We then describe the themes identified from the reflective interviews conducted during the first and final advisory group meetings (Aim 2).

### Aim 1: how parents’ involvement shaped the home–school partnerships study over time

Many changes to the home–school partnerships project occurred due to advisory group meeting discussions and feedback (see Figure 1). Key modifications to the methods included changing/editing parent interview survey questions to ensure accurate and meaningful translation of questions from English. For example, in the Vietnamese parent interview survey, the terms ‘strategies/support’, ‘interaction’, ‘barriers’ ‘and ‘in an ideal world’ did not easily or meaningfully translate into Vietnamese so were replaced by ‘tactics’, ‘communication and connection’, ‘difficulties’ and ‘power to change things’, respectively. Modifications were also made to interview schedules to include additional questions considered relevant for their communities (e.g., asking about parents’ own educational experiences and any key differences they observed between their schooling experiences and their children's experiences in Australia). A further reason for editing interviews was to safeguard that questions posed were culturally sensitive (i.e., broadening a question about parental involvement in their child's education so as to not elicit guilt/judgement from parents about this).

Towards the end of the home–school partnerships project, the advisory group meetings had a similar impact on shaping the interpretation of the research. Theme and subtheme names were more appropriately defined and revised by parents to ensure accurate understanding and representation of data. For example, mother blame was explicitly identified in a stigmatisation/discrimination theme in the Vietnamese parent advisory group meeting, and Chinese parents wanted ‘ineffective leadership’ made clearer in a subtheme related to parents lacking confidence and trust in teachers. Parents were also integral in the generation of bespoke, practical research recommendations for both parents and teachers (e.g., suggesting specific book recommendations for school libraries and book weeks, such as The Trung Sisters, Hai Bà Trưng, Mulan, With the Light, etc.).

Parents also influenced aspects of the dissemination phase of the research, including how key recommendations could be shared with communities. While the original research dissemination plan (devised without CALD community input) was to translate the full research report into community languages, both groups rejected this approach. Instead, they each suggested that the most effective way to disseminate findings to their communities would be to produce a 1- to 2-page document, which would be easily shareable on social media. Sections on ‘myth-busting’ (with myths taken from parent interviews for respective communities) and frequently asked questions (FAQs) for parents around education (i.e., ‘What are my child's education rights?’ ‘How/when do you need to enrol your child in a school?’ ‘How should I prepare for meetings?’) were suggested for inclusion. Parents stated that this type of content would be more accessible to parents from their communities and would directly address autism stigma and support parental advocacy in relation to their autistic children's education.

### Aim 2: understanding co-production partners’ (i.e., parents’) experiences of being involved in the home–school partnerships project over time

**Figure 2.**
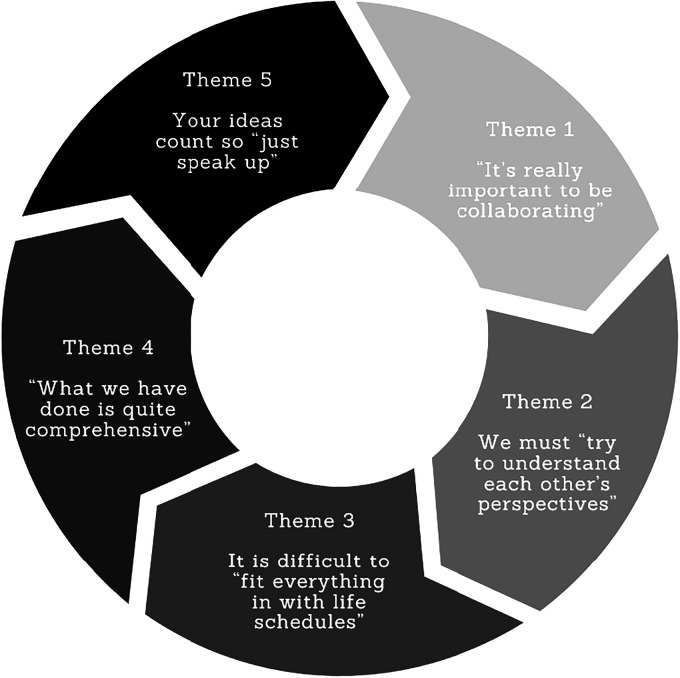
Themes arising from parents’ responses to questions.

We identified five key themes from our longitudinal analysis of the first and final advisory group meetings for the home–school partnership study (see Figure 2). Illustrative quotes with community (i.e., Chinese [C] and Vietnamese [V]), parent ID (i.e., 001, 002, etc.) and time point (i.e., initial [T1] or final [T2] are detailed below.

### Theme 1: ‘it's really important to be collaborating’ [V003_T1]

Parents frequently stated how they were ‘excited to be part of this research’ [V002_T1] and appreciated that their own ‘community [was] being respected’ [C004_T1]. They were specifically enthused about the research topic, namely parent–school partnerships for Chinese and Vietnamese parents of autistic children: ‘I was very excited and delighted to see the nature of the research which is to dig into something deeper in this group of parents’ [C001_T1]; ‘I’m so excited that there are areas like you who are looking into our backgrounds, especially the care for the family, for the Vietnamese’ [V003_T1]. There was likewise enthusiasm that their own personal experiences would be valuable for the project: ‘I sat down [and] I read your emails in detail. I felt a little bit excited about everything you put in and also those questions. I feel like I’m the right person for this’ [C002_T1]. Parents were also eager to be involved in the project as the topic resonated with their own interests: ‘I’m happy to be part of this … my passion is about disability, autism’ [V003_T1].

Underscoring the value of meaningful collaboration, parents were especially keen to ‘share [their] knowledge with other parents’ [V002_T1] and to ‘provide some relevant information’ [V003_T1]. Parents described the importance of partnerships and shared learnings: ‘I’m very grateful to be part of [the research] and I think it's really important to be collaborating and be willing to work with you’ [V003_T1]; ‘I feel I’m quite happy, actually very excited to contribute some thoughts and ideas to this research’ [C002_T2]. They also clearly outlined the potential benefits of cooperation, benefits they openly defined at the end of the project. Parents described how the learnings from the broader study ‘would greatly help others in improving their understanding in knowing what to do’ [C003_T2] and the ‘knowledge and understanding [would] improve education for the future generations’ [V003_T2].

### Theme 2: we must ‘try to understand each other's perspectives’ [C004_T2]

Parents often commented on how learnings could only occur through hearing divergent opinions, such as listening to ‘the different perspective of parents’ [C001_T1]. This view explained why parents wanted to hear experiences not just from their own communities – ‘Chinese parents want to know about Chinese parents’ experience’ [C003_T1] – but from other cultures and societies – ‘I’ve always wanted to know what challenges parents are facing from different cultures or different countries as well … a Sudanese background or an African background, are all parents with children with disability experiencing the same thing?’ [V003_T2]. Parents stated that by hearing diverse opinions you can ‘benefit from another parent's point of view’, by being exposed to things you have ‘yet to understand and haven’t yet experienced’ [V003_T1]. Parents described how ‘we all need to be open-minded to try to understand each other's perspectives’ [C004_T2], otherwise you can ‘just quickly go to judge people before even thinking about what's going on’ [V001_T2].

Nonetheless, parents recognised that sharing their stories was difficult. One parent commented: ‘When we recall what happened previously, things that happened at schools or troubles or anything, those details, we may struggle or feel heartbroken’ [C002_T1]. Similarly, another parent said: ‘With kids with special needs it sometimes gets very personal and then in meeting environments, like this, it can get really emotional, that's why I said to myself don’t get too emotional’ [V002_T2]. They reflected more broadly on how parents from their respective cultures struggled to voice their opinions for a variety of reasons. One parent said ‘Chinese parents are a bit shy … you need to have enough mental strength to go over your hurdle … [to be] upfront, with no hesitation’ [C003_T2]. Another parent also reflected that it was challenging for Vietnamese parents to be open because ‘they’re afraid that they will be judged, or their child will be judged’ [V002_T2].

### Theme 3: it is difficult to ‘fit everything in with life schedules’ [V002_T1]

Parents were further concerned about how they would balance participating in the research process alongside their other responsibilities. They noted how parents of children with disabilities ‘have to prioritise everyday tasks’ [C002_T1] and that ‘[organisation] for parents with children with disability is a big challenge’ [V002_T2]. Some parents explained how they had previously had to discontinue non-essential activities due to competing priorities. One parent described how she had to stop volunteering as she ‘couldn’t do it ongoing because [she's] got three kids with special needs’ [V002_T1]. One parent said that because she was ‘quite busy’ she did not manage to ‘really look at’ [C002_T1] the information emailed about the advisory group prior to the first meeting.

### Theme 4: ‘what we have done is quite comprehensive’ [C003_T2]

At the end of the process, parents appeared surprised at the scope of the broader home–school partnerships study on which they were co-production partners. They were impressed about how the broader project (including their contributions as advisory group members) had collectively managed to ‘gather all the opinions, all the recommendations’ [C004_T2]. Parents were also impressed by the depth of the data collected, and felt that the sensitive implementation of the study had allowed parents ‘a lot of time and courage to actually express their true feelings’ and so we had ‘real interview(s) from parents with the special needs family … real results to analyse and make the recommendations’, declaring that it was ‘an amazing result’ [V003_T2]. Another commented: ‘Basically [we] have everything there, evidence to be able to support what we’ve been discussing’ [V002_T2].

They noted that the amount of involvement from parents themselves on the broader home–school partnerships study was more than they had anticipated, ‘[collaborating in the process] was more complex, and more interesting’ [V002_T2]. Perhaps because of the scope and quality of the data collected, as well as their level of involvement, the importance of research impact following the home–school partnerships study emerged more strongly for parents over time: ‘Now it's more important how we’re going to make a difference’ [V001_T2]. And, whilst parents were clear about what they wanted from the home–school partnerships research – such as, ‘to improve public understanding of their rights around disability standards’^1^ – they also acknowledged that ‘implementation’ will be the ‘difficult part’ as ‘knowing the process and implementation are two different things’ [C003_T2].

### Theme 5: your ideas count so ‘just speak up’ [V002_T2]

Initially, there was some trepidation that parents’ voices would not be valued in the home–school partnerships study and may even be judged: ‘I hope that, you know, my ideas would count and what I’m sharing is right’ [V002_T2]. But, by the end of the study, parents understood that the research was being shaped by their opinions and their voices were catalysts for change. They stated that the home–school partnerships research gave ‘the opportunity for us as the ethnics or from Vietnamese backgrounds to be able to raise our opinions’ [V003_T2] and they hoped ‘more people could hear our voices’ [C002_T2]. Despite noting the high levels of involvement required of the project, parents also described wanting to be *more involved* in the home–school partnerships research process: ‘I wish I would be able to participate in the interviews [for the home–school partnerships study] so then I’d be able to see [parents’] emotions’ [V003_T2].

Parents also spoke of how much they themselves had learnt throughout the process. One mother said that throughout the ‘whole journey’, she had ‘really learnt a lot from the other parents’ [C005_T2]. Another mentioned how she had also ‘learned a lot of things which [she] took for granted sometimes’ [V002_T2]. This shared knowledge, coupled with feeling as if their voices were heard, appeared to translate into increased confidence and wellbeing outside of the research project. One parent explained that what she had learnt through the home–school partnerships research process had taught her to be ‘more firm’ when speaking to ‘the school and professionals’ [C001_T2]. Another parent stated: ‘The one thing that I have thought throughout this … I’ve never asked myself if I’m okay or is this too much for me to handle?’, finishing by saying ‘If you need help, just speak up’ [V002_T2].

## Discussion

Co-produced research is vital for ensuring research with marginalised and/or minority communities is conducted in a respectful, responsive and ethical manner (Belone et al., 2016). Including diverse research partners in the research process also allows research recommendations to be more easily generalisable to autism and autistic communities more broadly (Cascio et al., 2020; Mallipeddi & VanDaalen, 2021). Here, we report on Chinese and Vietnamese parents’ involvement as community partners in a research project focusing on home–school partnerships for autistic children from CALD backgrounds. We sought to understand how parents’ involvement shaped the home–school partnerships study, as well as their experiences of the participatory process.

Through existing community connections, we recruited a diverse group of Chinese and Vietnamese parents to this broader project, none of whom had been involved previously in research as a community partner. Parents attended four advisory group meetings over the course of the year-long home–school partnerships study alongside researchers and professionals. Parents’ involvement in the advisory group meetings for the home–school partnerships study directly shaped the research itself, from determining final parent interview schedules to generating key recommendations from the research findings. Parents themselves reported being keen to collaborate and to hear and share opinions for the purpose of the home–school partnership study – although they noted how emotionally difficult sharing their stories could be. While parents had some initial concerns about being involved as a community partner, these concerns subsided over the course of the project. Through hearing others’ stories and sharing their own in advisory group meetings, parents reported ancillary benefits of their involvement, including increased self-advocacy and wellbeing.

### The impact of parents’ participation

Perceptions about disability are interwoven with language, with culture moderating and likely perpetuating pejorative views about disability (Altarriba & Basnight-Brown, 2022). Community checking of translations is therefore an important but often undervalued activity (Taibi & Ozolins, 2016), which involves checking for semantic equivalence (i.e., ensuring that terms reflect the same construct in different languages; Chang et al., 1999) as well as pragmatic and socio-linguistic equivalence and acceptability (Taibi & Ozolins, 2016). In the context of autism research, whilst the word ‘autism’ can be directly interpreted into Vietnamese, in Chinese autism literally translates to ‘lonely disease’ (Su et al., 2019) so care needs to be taken in how the condition is understood by the community. For the success of the broader home–school partnerships project, parents’ ongoing involvement was vital in safeguarding that the research measures and approaches translated accurately and sensitively cross-culturally (Freeth et al., 2014).

Another direct impact of parents’ involvement in the home–school partnerships study was towards the end of the project, through shaping the approach to community research dissemination. Previous studies with marginalised communities have similarly reported how community involvement meaningfully influenced community dissemination – both in delivery and message (Kildea et al., 2009). The proposed dissemination plan suggested by parents (brief, easily shareable flyers) ensured that communities could access and share findings about the home–school partnerships study on social media more easily than the research team's initial suggestion (De las Nueces et al., 2012; De Souza et al., 2021; Wild et al., 2021). The suggested inclusion of FAQs and information debunking community-specific myths about autism, alongside the key research fundings, was also intended to serve as a useful mechanism to address autism stigma in their communities (Ha & Whittaker, 2022; Liu & Fisher, 2017). This ancillary benefit of challenging autism stigma through the participatory process with CALD communities has previously been reported (Aabe et al., 2019). Our findings strengthen the evidence of the myriad proximal and distal benefits of conducting autism research in partnership with CALD communities and further reinforce the need for funders to prioritise this type of research (West et al., 2016). However, prioritising and delivering outcomes desired by funders, participatory partners and researchers within the research budget and timeframe was challenging; hence, extra time and money for production of community-specific outputs should be considered in budgets.

### Parents’ experiences

Our parents reported feeling valued they were being heard by others during the home–school partnerships advisory group meetings, but they also found that sharing their experiences took its toll. Other non-CALD partners in the fields of autism and health research have described similar experiences (Pellicano et al., 2021; Pinto et al., 2013). Where research is undertaken by people with comparable experiences to those of the participants being studied – in this instance, parents from CALD communities who have autistic children – there is more potential for harm during the research process since the topic is inherently more personal than for researchers who may not have lived experience (Banks et al., 2013). Within co-produced autism research studies, it is imperative that trusting relationships are built and maintained, and that there are clear ground rules from the start of projects to foster and uphold a safe research environment (Aabe et al., 2019; Stark et al., 2021).

Previous participatory research with autistic adults has reported that the process of sharing their stories led to an increased sense of agency and wellbeing (Pellicano et al., 2021). Here, our parents also reported personal growth, self-advocacy and self-acceptance because of their involvement in the study. Knowing that parents of autistic children often experience poorer mental health and wellbeing (Green et al., 2021), involvement in research could serve as a means of support for parents themselves. Knowing that there may be broader, unseen benefits for parents could be one mechanism through which to increase broader community involvement in autism research – although the benefits of such engagement for these parents should continue to be evaluated, given that the extent and nature of community involvement can vary widely.

The challenges of prioritising and having limited time emerged as potential barriers for parents at the beginning of their involvement in the project – an issue that has also been noted in other co-produced research (Pinto et al., 2013). Despite these concerns, all of our parents attended all advisory group meetings, even when past longitudinal participatory research has reporting a dwindling of meeting attendance over time (Pinto et al., 2013). Our parents were in fact surprised by their levels of involvement and the scope of the study and the breadth and depth of the knowledge that was co-learnt and co-created (Amauchi et al., 2021).

There are likely to be many factors contributing to parents’ sustained and collaborative involvement here. First, parents were recruited via existing relationships with individual researchers and our partner, Positive Partnerships. Second, we sought to ensure flexibility around meeting logistics (e.g., meeting times, language use) and remunerated them for their contributions to knowledge production aligning with core values of supportive infrastructure in participatory research (Fletcher-Watson et al., 2019). Finally, we also ensured that parents did not feel ‘outnumbered’ (Jose et al., 2020) through having equal or greater numbers of parents to researchers and professionals. Building relationships, flexibility, valuing knowledge and reducing power imbalances have all previously been reported as core principles of co-produced research (Aabe et al., 2019; Hickey et al., 2018) and we sought to adhere to those principles in this study.

### Limitations and future directions

Notwithstanding, our study has several limitations. First, we acknowledge this study is limited by parents’ views of being community partners being collected by researchers known to them, but hope that the findings are still an important step in understanding how we can include diverse community partners in research and the impact of their involvement. Relatedly, there may have been value augmenting the group-based feedback session with individual interviews (especially with an impartial interviewer) to allow parent advisors to share their experiences in another forum. Future research should consider the best mechanism (and associated costs) for collecting anonymous feedback from participants to mitigate potential positive biases. Second, the experiences of parents recruited into our advisory groups may not reflect the opinions or experiences of newly arrived migrants, especially as our parent advisors were mostly comfortable communicating in English. That said, most of our parent advisors were new to research, and all were new to co-produced research, suggesting that we accessed a group of parents who may have not yet been heard during the research process. Since parents felt less confident about being involved in the project at the beginning, understanding how effectively to prepare and upskill new community partners to the research process is an important avenue for future research.

## Conclusion

The Chinese and Vietnamese parents who co-produced our study taught us a great deal, especially about approaches to meaningfully share findings with different communities. They also felt that they personally benefited from their involvement in the study. These findings contribute to an understanding of the benefits and challenges of participatory autism research for both communities and researchers. We hope that this study strengthens the evidence for the value of partnering with CALD communities and highlights potential challenges to be considered in future projects.

## Supplemental Material

sj-docx-1-dli-10.1177_23969415231210482 - Supplemental material for ‘It's really important to be collaborating’: Experiences of participatory research for Chinese and Vietnamese parents of autistic children Click here for additional data file. Supplemental material, sj-docx-1-dli-10.1177_23969415231210482 for ‘It's really important to be collaborating’: Experiences of participatory research for Chinese and Vietnamese parents of autistic children by Jodie Smith, Aspasia Stacey Rabba, Poulomee Datta, Emma Dresens, Rena Wang, Lin Cong, Ngoc Dang, Gabrielle Hall, Melanie Heyworth, Wenn Lawson, Patricia Lee, Rozanna Lilley, Emily Ma, Hau T T Nguyen, Kim-Van Nguyen, Phuc Nguyen, Chong Tze Yeow, and Elizabeth Pellicano in Autism & Developmental Language Impairments

## Notes

1. Education providers’ legal responsibilities.
