# Supplementary material for: ‘It's really important to be collaborating’: Experiences of participatory research for Chinese and Vietnamese parents of autistic children
Source: Autism Dev Lang Impair. 2023 Nov 13;8:23969415231210482. doi: 10.1177/23969415231210482 (PMC10644728; doi:10.1177/23969415231210482)
Supplement: sj-docx-1-dli-10.1177_23969415231210482 - Supplemental material for ‘It's really important to be collaborating’: Experiences of participatory research for Chinese and Vietnamese parents of autistic children [file sj-docx-1-dli-10.1177_23969415231210482.docx]

Supplementary Table 1. Participatory research principles and activities adopted in the current study based on previous co-produced autism studies (Aabe et al., 2019; Fletcher-Watson et al. 2019).

| **Current Study** | **Principles** |
| --- | --- |
| We selected communities where researchers had established connections so parents (community partners) could be recruited by someone known and trusted to them, in their home language where appropriate. We therefore maintained and developed relationships with community organisations (i.e., the Chinese Parents Special Support Network and Extended Families [supports for Vietnamese families with children with a disability]). | - Establishing effective partnerships - Building and maintaining relationships with organisations and communities - Empathy |
| We collected information on preferred language (written and spoken) and had interpreters in every meeting and encouraged sharing of information in languages parents felt most comfortable communicating in. | - Power-sharing - Inclusion of all perspectives and skills |
| Parents were collaborators from the project outset and involved in the study design, implementation and dissemination with key decisions jointly agreed as a group. | - Inclusion of all perspectives and skills - Valuing knowledge of all partners - Authenticity |
| The first meetings involved discussing what co-production involved, namely researchers and community members working together on a project where both the skills of researchers and the expertise that people on the autism spectrum and their families/carers have gained through their lived experience are equally valued. | - Respect - Assumptions - Power-sharing |
| Parents were remunerated for both their indirect (i.e., preparing for meetings) and direct (i.e., attending meetings) contributions. | - Infrastructure - Valuing knowledge of all partners |
| Meetings were held at a convenient time for parents (i.e., during evenings/lunchtimes) so that all parents could attend. | - Infrastructure - Valuing knowledge of all partners |
| To ensure parents did not feel like they were a ‘token’ advocate on behalf of their communities, we ensured we had 3-5 parents from each community (equal to or exceeding the number of researchers/professionals) so that divergent opinions and aspects of intersectionality could be considered in the meetings. | - Respect - Assumptions - Authenticity - Power-sharing - Inclusion of all perspectives and skills - Valuing knowledge of all partners |
| Parents were sent information after each meeting outlining the key changes that were made to the research process based on discussions in advisory groups. The intention was to be clear that all perspectives were considered and parents held power in the decision-making process. | - Authenticity - Valuing knowledge of all partners |
| Parents are co-authors on papers generated from the broader study [WITHHELD FOR BLIND REVIEW] and also co-authors on the current study allowing for development of other research skills. | - Developing skills, capacity and opportunities for personal growth |
